# Contribution of voluntary fortified foods to micronutrient intake in The Netherlands

**DOI:** 10.1007/s00394-021-02728-4

**Published:** 2022-01-01

**Authors:** Marjolein H. de Jong, Eline L. Nawijn, Janneke Verkaik-Kloosterman

**Affiliations:** National Institute for Public Health and the Environment (RIVM), Bilthoven, The Netherlands

**Keywords:** Voluntary food fortification, Micronutrients, The Netherlands, Habitual intakes

## Abstract

**Purpose:**

In the Netherlands, voluntary fortification of foods with micronutrients is allowed under strict regulations. This study investigates the impact of voluntary food fortification practices in the Netherlands on the frequency and type of fortified food consumption and on the micronutrient intakes of the Dutch population.

**Methods:**

Data of the Dutch National Food Consumption Survey (2012–2016; *N* = 4314; 1–79 year) and the Dutch Food Composition Database (NEVO version 2016) was used. To determine if voluntary fortified foods could be classified as healthy foods, criteria of the Dutch Wheel of Five were used. Habitual intakes of users and non-users of voluntary food fortification were calculated using Statistical Program to Assess Dietary Exposure (SPADE) and compared.

**Results:**

Within the Dutch population, 75% could be classified as user of voluntary fortified foods. Consumed voluntary fortified foods were mostly within food groups ‘Fats and Oils’, ‘Non-alcoholic Beverages’ and ‘Dairy products and Substitutes’ and fell mostly outside the Wheel of Five. Voluntary foods contributed between 9 and 78% to total micronutrient intake of users. Users had up to 64% higher habitual micronutrient intakes, compared to non-users. These higher intakes resulted into lower risks on inadequate intakes, and did not contribute to increased risks of excessive intakes.

**Conclusion:**

Although voluntary fortified foods increased micronutrient intakes, most of these foods cannot be classified as healthy foods. Future studies should study the association between higher micronutrient intakes and (potential) excessive intakes of e.g. saturated fat and sugar to better understand the role of voluntary fortified foods in a healthy food pattern.

**Supplementary Information:**

The online version contains supplementary material available at 10.1007/s00394-021-02728-4.

## Introduction

Micronutrients (vitamins and minerals) are essential for health [1]. In the ideal situation, micronutrient intakes of the population are within the boundaries of inadequate and excessive intakes. However, in many countries, including the Netherlands, at least for some micronutrients the intakes are suboptimal [2].

A strategy to improve the micronutrient intake in a population is food fortification, although in the Netherlands, the advice of dietary supplement intakes is more common to improve micronutrient intakes of specific subgroups. Micronutrients are mandatory or voluntarily added to (specific) foods. With legislation, fortification can be used to successfully prevent both too low and excessive micronutrient intakes. Within the European Union, it is regulated which micronutrients may be added to foods and in what form. For example, unprocessed foods may not be fortified. However, maximum fortification levels have not been determined yet [3]. Consequently, European member states, including the Netherlands, have additional national legislation. In the Netherlands, mandatory fortification is legally not feasible, however, some fortification is encouraged by covenants between industry and government [4]. See Textbox 1 for the full overview of the legislation for food fortification in the Netherlands. To prevent excessive vitamin A, D, folic acid, iodine, selenium, copper and zinc-intakes, it is prohibited to fortify foods with these micronutrients. There are, however, specific exemptions for food fortification with these nutrients, under specific conditions, as described in Textbox 1 [4]. In addition, Dutch legislation specifically allows adding all micronutrients to foods for restauration or substitution purposes. This is allowed to restore the naturally present micronutrient content lost during processing (restauration), or to replicate micronutrient content of similar foods (substitution).

Although micronutrient intake is important, healthy foods should not only provide the essential nutrients, but should also be low in salt, sugar, trans- or saturated fats [5]. It is, therefore, important to investigate what type of foods are voluntarily fortified, how often these are consumed and if these foods can be considered as healthy.

Voluntary food fortification can only be prohibited if it poses a risk on excessive intakes among the Dutch population. Voluntary food fortification policy in the Netherlands is, therefore, only established to assure safety rather than health. However, as micronutrient intake in the Dutch population is low for vitamin B2, B6, C, D and folate among different population groups, voluntary food fortification might have an impact in improving intakes among users [6].

The aims of this study were to evaluate the consumption of voluntarily fortified foods in the Netherlands and to assess the contribution of these foods to the adequacy and safety of micronutrient intake. An additional aim is to study potential differences in characteristics between users and non-users of voluntarily fortified foods, as well as the classification of fortified foods as healthy foods.

Textbox 1: legislation for food fortification in the Netherlands Micronutrients may be added to foods with a minimum of 15% and a maximum of 100% of the reference intake for micronutrients [4].

Food fortification with vitamin A (retinoid form), vitamin D, folic acid, selenium, copper and zinc is allowed for restauration or substitution purposes only [4]. It is not obligated to declare these additions on the ingredient list. Exemptions for fortification with vitamin A, D, folic acid, iodine and zinc exist:

- Fortification of margarines and other plant-based fats with vitamin A (retinoid form) and D is encouraged by a covenant between the margarine industry and Dutch authorities [4, 7]
  ▪ With a maximum vitamin A content of 8 µg RE and maximum vitamin D content of 0.075 µg/g product [4]
  ▪ Specific margarines and other spreadable fats intended for persons older than 60 years old may contain at least 0.2 µg and maximum 0.25 µg vitamin D/g product [8]
- Iodized salt with high iodine content (up to 65 mg/kg salt) is allowed in bakery products and also encouraged by a covenant [4, 9]
  ▪ Other foods may be produced with iodized salt with a lower iodine content (up to 25 mg/kg salt)
- Folic acid and vitamin D are allowed to be added up to 100 µg and 4.5 µg per 100 kcal product, respectively [10]
- For zinc, product-specific exemptions were made [11, 12]

## Methods

### Survey population

Data on food consumption of the Dutch population were collected in the Dutch National Food Consumption Survey (DNFCS) 2012–2016 (*N* = 4313, 1–79 year.). The DNFCS 2012–2016 is in detail described elsewhere [6]. In short, a sample representative for the Dutch population was selected from a consumer’s panel (Kantar TNS; net response rate 65%). Exclusions for participating were pregnancy and giving breastfeeding, as well as living in an institution. Also, a sufficient command of the Dutch language was required.

Two 24-h recalls were collected on non-consecutive days to estimate food consumption, using GloboDiet (IARC^©^; former EPIC-Soft). Trained dieticians performed the interviews and a distinction was made for different ages. For children aged 4–15 years old, interviews took place at home together with a parent or caretaker. For younger children, the parents or caretakers were interviewed about their child’s food consumption. For participants between 16 and 70 years old, the interviews were unannounced and performed by phone. Besides the 24-h recalls, also a food diary was used for the youngest (1–8 years) and oldest (71–79 years) age groups. Also, for the 71–79 year olds, these interviews were performed at home. To estimate the micronutrient intake, the food consumption data was linked to the Dutch Food Composition Database (NEVO; NEVO-online version 2016/5.0, RIVM, Bilthoven, 2016, with additions for the DNFCS 2012–2016).

The age at the first 24-h recall day was used as the age of the respondent. On the first recall day, height and weight were measured for children up to 15 years and participants aged 71–79 year old. For all other ages, height and weight was self-reported. The body mass index of the participants was calculated as the bodyweight divided by the height squared (kg/m^2^) and categorised into (extremely) underweight (BMI < 18.5 for adults), normal (BMI 18.5–25 for adults) and overweight/obesity (BMI > 25 for adults). For children the same categories, but different age-specific cut-off values were used. With a general questionnaire, other general information of the participants was collected, including information on various background factors, such as educational level, working status, native country, family size, various life style factors, such as patterns of physical activity, smoking and use of alcoholic beverages and various general characteristics of the diet, such as special diets and eating habits. Taking into account the different way of living, the questionnaires differed among different ages. The degree of urbanisation was categorised in extremely urbanised (2500 or more addresses/km^2^), strongly (1500–2500 addresses/km^2^), moderately (1000–1500 addresses/km^2^), hardly (500–1000 addresses/km^2^) and not urbanised (fewer than 500 addresses/km^2^). The educational level concerned the highest completed educational level of the participants or, in case of participants ≤ 18, of the head of household.

### Definition voluntary fortified foods

Voluntary fortified foods were defined by the presence of micronutrients in the ingredient list on the food label, under specific conditions:

- As micronutrients added for restauration or substitution purposes or added as anti-oxidant are mostly not mentioned in the ingredient list, these nutrients were not included as fortification in this study. If they were for some reason included in the ingredient list, they still were included as food fortification.
- Only foods regulated within the European legislation on the addition of vitamins and minerals and of other substances to foods were included [3]. This means specific foods intended for infants and young children, including young child formulae, foods for special medical purposes and foods for total diet replacement for weight control, for which specific legislation applies, were not considered as voluntarily fortified foods.
- Only voluntary fortified foods were included in this study, meaning foods for which fortification was encouraged with a covenant, including vitamins A and D in margarines and other plant-based fats and iodine added to bakery salt were not included as fortified foods. Other micronutrients voluntarily added to these types of fats, however, were included as fortification in this study.

Nutrient content of fortified foods was assumed to be equal to the amount of micronutrient added to the foods and declared on the food label.

Users of voluntary fortified foods were identified as those consuming at least one voluntary fortified food product on at least one of the recall days. Non-users were identified as those who did not consume any fortified food products on both recall days. In this study, we only considered the micronutrient intake from foods, intake from dietary supplements was excluded.

### Variation in- and amount of voluntary fortified foods consumed

To study how many different types of fortified foods were consumed on a recall day within the Dutch population, voluntary fortified foods were distinguished by their NEVO-code. Within NEVO, all details of same types of foods with comparable compositions are merged within one NEVO-code [13].

To investigate how often a voluntary fortified food was consumed on a recall day, all consumed fortified foods were counted, also if the same type of food was consumed on multiple mealtimes. A mealtime can be defined as moment a participant ate a meal or snack on a recall day. If the same type of voluntary fortified food was consumed during the same mealtime, it was only counted once.

It is also possible that users consumed the same voluntary fortified food on multiple mealtimes a day. To investigate how often these foods were consumed on a recall day, the amount of consumed voluntary fortified foods by each user on a recall day was calculated. Within the same mealtime moment, the consumption of multiple portions of the same voluntary fortified foods (based on the same NEVO-code) was only counted once. If, however, the same voluntary fortified foods was consumed within for example two different mealtimes (e.g. breakfast and lunch), it was counted twice.

### Food groups of the voluntary fortified foods

Voluntary fortified foods were classified according to the Globodiet food groups. These consist of 18 main- and 78 subgroups. Of these subgroups, 16 were divided into a total of 55 sub-subgroups. As fortification is not allowed for unprocessed foods [3], not all (sub-) groups were included in current study.

### Wheel of Five

The Wheel of Five is a practical nutrition information tool, created by the Dutch Nutrition Centre [14]. The Wheel of Five helps to guide the Dutch population to a healthy consumption pattern with sufficient nutrients. Foods are categorized as falling in- or outside the Wheel of Five. The foods falling inside the Wheel of Five can be considered as foods fitting within a healthy food consumption pattern, based on the Dutch dietary guidelines 2015 of the Health Council of the Netherlands [15]. The Wheel of Five consists of five food groups: ‘Fruit and vegetables, ‘Spreadable fats and cooking fats’, ‘Fish, legumes, meat, eggs, nuts and dairy’, ‘Bread, cereals and potatoes’ and ‘Drinks’. Foods within these five food groups are categorized as healthy options (within the Wheel of Five) and less healthy options (outside the Wheel of Five). Foods falling outside the Wheel of Five have in general a too high content of salt, sugar, trans- or saturated fat or a too low fibre content. Foods fitting in food groups, other than the five mentioned above, fall outside the Wheel of Five (see Online Appendix 1 for all foods consumed in DNFCS 2012–2016 falling in- and outside the Wheel of Five). In the current study, the consumed fortified and non-fortified foods were classified according to the conditions of the Wheel of Five.

### Assessment of risk on inadequate and excessive micronutrient intakes

The risk on inadequate and excessive micronutrient was assessed using the dietary reference values established by the Health Council of the Netherlands [16, 17]. For vitamin D, both the reference value assuming sufficient as the value assuming insufficient sunlight exposure was used to compare with the habitual intake.

### Statistical analysis

Due to the study design of the DNFCS 2012–2016, children were overrepresented in the sample to obtain equal age groups [6]. To assure representativeness with the Dutch population (representative for calendar year 2014), a weighting factor was applied in the analysis. Within this weight factor socio-demographic factors, season and day of the week were included. Compared to the non-weighted results, deviations were small, therefore, only the weighted results were presented.

General characteristics of users and non-users were compared with a Chi-Square test. A *p* value < 0.05 was considered to indicate a statistically significant difference between the groups. The *p* values were corrected for multiple comparisons using the Bonferroni correction [18].

The proportion of users of foods within the Globodiet food(sub-)groups was calculated by dividing the amount of recall days a voluntary food was consumed by the total recall days in the DNFCS 2012–2016 (*n* = 8626). Also, the consumed foods within the (sub-)groups (in g/day) was divided by the total consumed foods within both the subgroup as the group (in g/day). The amount of consumption within a (sub-)food group on a recall day was assessed by calculating the consumption (P5, P50, P95) of users only.

The contribution of voluntary fortified foods and the habitual intakes were calculated for the micronutrients for which voluntary fortification was allowed: calcium, iron, magnesium, phosphorus, potassium, zinc, vitamin A (as retinol activity equivalents (RAE)), vitamins B_1_, B_2_, B_3_, B_6_, B_12_, C, D and E, and total folate. Vitamin A in RAE was calculated as μg retinol + β-carotene/12 + μg other carotenoids/24 [17]. Folic acid is the synthetic form of folate, added to fortified foods. Total folate was expressed as folate equivalents and was calculated using the amount of folate naturally present in foods + 1.7 times folic acid from enriched foods (in μg) [13].

Contribution of voluntary food fortification to total micronutrient intake was calculated for each recall day a voluntary fortified food was consumed. We, therefore, did not calculate the average intake over 2 recall days, or the habitual intakes for this analysis, as some subjects did not consume the same foods on both the recall days. For these users, the other recall day was not included in the analysis for contribution to total micronutrient intake.

Habitual intake (also referred to as usual intake) distribution and 95%-confidence intervals (CI) of users and non-users of foods fortified with that specific micronutrient was estimated by correcting the data for the within-person variation using the Statistical Program to Assess Dietary Exposure (SPADE version 4.0.85 of 16 December 2020). Intakes were age-dependently modelled, using the 1-part model. These analyses were performed in R version 1.1.383. Due to too small user groups, no habitual intake could be calculated for zinc. The habitual intakes were calculated within four age–sex classes: boys 1–17 year old, girls 1–17 year old, men 18–79 year old and women 18–79 year old. The habitual intake distribution of users of voluntary fortified foods were compared to those of non-users.

For micronutrients with an ‘adequate intake’ (AI) set, the adequacy was qualitatively assessed. Habitual median intakes above the AI were considered as a low risk of inadequate micronutrient intake. If the median habitual intake was below the adequate intake, no statement about the adequacy could be made. Evaluation of the risk on inadequate intake for micronutrients with an ‘estimated average requirement’ (EAR) set, was performed using the EAR cut-point method [19]. Here, the proportion of the population with habitual intakes below the EAR was estimated. Also, the proportion of the population with a habitual micronutrient intake above the upper level (UL) were estimated.

Comparisons in habitual intakes and proportions below the EAR and above the UL of users and non-users were performed by estimating the difference between users and non-users and calculating 95%-confidence intervals for these differences, based on 200 bootstrap iterations, similar to Dekkers and Slob [20]. When the CI for difference did not include zero, habitual intakes or proportions below or above EAR or UL were considered as statistically different between users and non-users.

For magnesium, the group of non-users was too small (*n* = 74) to calculate a 95%-CI. Therefore, it was not possible to assess if intakes were significantly different between users and non-users or to compare with the dietary reference values.

Unless otherwise stated, statistical analyses were performed using SAS version 9 (Windows version 6.3.9600).

## Results

### Characteristics of the study population

Three quarters of the Dutch population can be considered as a user of voluntary fortified foods (Table 1). Among the users of these foods there were significantly more children, people with a normal or low BMI and people using fortified margarines and other plant-based fats, compared to the non-users.

**Table 1:** Characteristics of users of fortified foods excluding the use of margarines and other plant-based fats fortified with vitamin D and retinol

	Non-users (*n* = 1074)^a^	Users (*n* = 3239)^a^	Adjusted *P *value^b^
Sex			1.000
Men	559 (52%)	1606 (50%)	
Women	515 (48%)	1633 (50%)	
Age			< 0.0001
Children (1–17 years)	86 (8%)	781 (24%)	
Adults (18–79 years)	988 (92%)	2459 (76%)	
BMI^c^			0.008
(extremely) Underweight	19 (2%)	110 (4%)	
Normal weight	430 (43%)	1524 (50%)	
Overweight/obesity	543 (55%)	1390 (46%)	
Smoking^d^			1.000
Yes	203 (21%)	569 (23%)	
No	778 (79%)	1854 (77%)	
Alcohol user^d^			1.000
Yes	714 (72%)	1819 (74%)	
No	274 (28%)	640 (26%)	
Fortified margarine and other plant-based fat user			< 0.0001
Yes	686 (64%)	2940 (91%)	
No	387 (36%)	299 (9%)	
Following a diet			0.1092
Yes	182 (17%)	398 (12%)	
No	891 (83%)	2841 (88%)	
Sports			1.000
Yes	455 (46%)	1229 (51%)	
No	526 (54%)	1194 (49%)	
Days a week 1 h activity			1.000
3 or less	15 (27%)	80 (27%)	
4 or 5	8 (14%)	44 (14%)	
6 or 7	33 (60%)	178 (59%)	
Educational level^e^			1.000
Low	277 (26%)	776 (24%)	
Middle	431 (40%)	1409 (43%)	
High	366 (34%)	1054 (33%)	
Urbanisation^f^			1.000
Extremely/strongly	523 (49%)	1537 (47%)	
Moderately	192 (18%)	665 (21%)	
Hardly/not	358 (33%)	1037 (32%)	
Ethnicity			1.000
Dutch	978 (91%)	2991 (92%)	
Western immigrant	31 (3%)	79 (2%)	
Non-Western immigrant	62 (6%)	169 (5%)	
Season (First recall day)			0.512
Spring	276 (26%)	803 (25%)	
Summer	236 (22%)	843 (26%)	
Autumn	312 (29%)	766 (24%)	
Winter	250 (23%)	828 (26%)	
Recall days			1.000
Weekend/week	518 (48%)	1595 (49%)	
Only week	372 (35%)	1051 (32%)	
Only weekend	184 (17%)	592 (18%)	

Weighted for socio-demographic factors, season and day of the week

^a^Not all characteristics were collected for all participants and as a result of the use of a weight factor, results needed to be rounded to numbers without decimals, resulting into some groups of non-users and/or users with an n not equal to 1074 and/or 3239

^b^*P* values calculated with the Chi-square test, based on the weighted values and corrected with the Bonferroni correction

^c^For children age-specific cut-off values were used. For adults: (extremely) underweight: BMI < 18.5, normal weight BMI = 18.5–25, overweight/obesity: BMI > 25

^d^For participants ≥ 18 years old

^e^The highest education of the parents for children

^f^Extremely/strongly urbanised: > 1500 addresses/km^2^, moderately urbanised: 1000–1500 addresses/km^2^, hardly/not urbanised: < 1000 addresses/km^2^

### Consumption of fortified foods

**Fig. 1:**
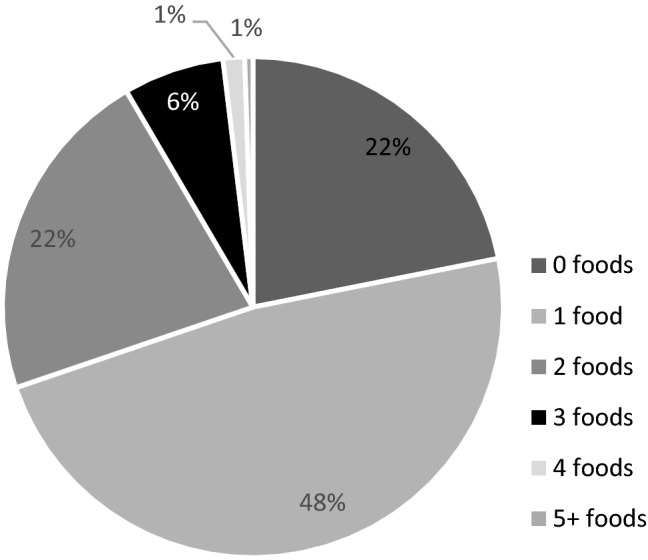
Distribution of the amount of consumed types fortified foods on a recall day within the Dutch population. Weighted for socio-demographic factors, season and day of the week

Fortified food users do not use fortified foods on every recall day. On less than a quarter of the recall days, users of fortified foods did not consume any fortified food (Fig. 1). On approximately half of the recall days, one type (based on the NEVO-code) of voluntary fortified food was consumed). On less than 2% of the recall days, ≥ 4 types of voluntary fortified foods were consumed, with a maximum of six types a day.

Some subjects consumed the same type of voluntary fortified food more than once on a recall day. On 42%, only one voluntary fortified food was consumed. On 16% and 7% of the recall days, 2 and 3 voluntary fortified foods were consumed, respectively, with a maximum of 17 foods.

### Food groups of the consumed fortified products

Mostly fortified foods were consumed from food groups ‘Fat and Oils’ (on 40% of all recall days), ‘Non-alcoholic beverages’ (on 20% of all recall days) and ‘Dairy products & substitutes’ (on 8% of all recall days; Table 2). The most frequent consumed fortified foods included sodas, fruit drinks and milk substitutes. Also, the consumed quantity of users on a consumption day was the highest for non-alcoholic beverages and dairy products: users of fortified dairy foods and non-alcoholic beverages used over 200 g of that food on a recall day (based on the median intake). Almost all milk substitutes consumed were fortified (91%). Also, a large proportion of the hot and cold-meat substitutes consumed were fortified (64–80%).

**Table 2:** Consumption of fortified foods within food groups and subgroups by the Dutch population aged 1-to-79 years (DNFCS 2012–2016), weighted for socio-demographic factors, season and day of the week

				On a consumption day (users of these fortified foods only)		
Food groups and subgroups	% fortified foods users within food (sub-) groups on a recall day	% consumed fortified foods within the total food group (based on g/d)	% consumed fortified foods within the total subgroup (based on g/d)	Median fortified food consumption users (g/day)	P5 fortified food consumption users (g/day)	P95 fortified food consumption users (g/day)
Dairy products and substitutes	8.4	4.9	–	206	48	545
Non fermented milk and milk beverages	0.0	0.0	0.2	222	101	375
Fermented milk, milk beverages and yoghurt	2.5	1.2	13.2	225	88	534
Milk substitutes and milk substitute products	2.7	1.5	90.6	224	50	808
Yoghurt	0.9	0.3	3.5	200	39	310
Fromage blanc, petits suisses	0.0	0.3	16.2	99	28	446
Cheeses (including spread cheeses)	0.2	0.0	0.3	14	8	38
Cream desserts, puddings (milk based)	0.7	0.2	4.8	198	76	352
Sorbet/water ice	0.3	0.3	13.1	45	32	53
Cereals and cereal products	3.6	1.1	–	30	11	102
Bread	0.5	0.3	0.4	54	23	153
Crispbread, rusks	0.2	0.1	0.8	19	7	75
Breakfast cereals	2.9	0.7	15.8	30	10	60
Meat, meat products and substitutes	1.5	1.3	–	61	9	151
Hot meat substitutes	1.1	1.1	63.8	77	13	162
Cold meat substitutes	0.4	0.2	79.6	11	6	31
Fats and oils	39.5	24.8	–	10	1	36
Unclassified and combined fats	4.1	2.5	19.7	5	1	19
Vegetable oils	0.2	0.1	0.6	6	1	22
Butter	0.2	0.1	0.9	6	1	23
Margarines and cooking fats	36.1	23.3	35.9	10	1	37
Other animal fats (including fish oils)	0.0	0.0	0.0	4	4	4
Sugar and confectionery	2.1	1.3	–	12	3	38
Jam, jelly and marmalade	0.2	0.2	1.2	35	11	91
Other sweet spreads	0.4	0.2	5.6	6	3	12
Syrup (incl. from can and for beverages)	1.1	0.7	36.3	12	4	27
Chocolate spread and chocolate powder	0.4	0.2	3.5	9	1	25
Confectionery non-chocolate	0.1	0.1	0.3	25	25	41
Cakes and sweet biscuits	2.8	3.1	–	38	13	73
Cakes, pies, pastries and puddings (non-milk)	0.1	0.1	0.2	28	25	66
Dry cakes, sweet biscuits	2.7	3.0	5.0	38	13	69
Non-alcoholic beverages	20.3	4.8	–	217	15	843
Fruit and vegetable juices	1.2	0.2	5.0	208	96	490
Carbonated-, soft-, isotonic drinks	19.2	4.6	17.4	216	12	850
Condiments, spices, sauces and yeast	0.9	1.1	–	11.6	1	45
Other and mixed sauces	0.7	0.6	3.5	24	2	46
Dressing sauces, mayonnaises, etc	0.1	0.0	0.2	10	4	37
Unclassified and combined condiments	0.2	0.0	0.4	1	1	4
Miscellaneous	0.1	0.7	–	48	17	117
Vegetarian products/dishes	0.1	0.7	100	48	17	117

*P5* fifth percentile, *P95* ninety-fifth percentile

### Wheel of Five

Most of the voluntary fortified foods consumed in DNFCS 2012–2016 fell outside the Wheel of five (Table 3). The proportion of both fortified as non-fortified foods falling inside the Wheel of Five was comparable for dairy and fats and oils. For cereals, however, the proportion non-fortified foods falling within the Wheel of Five was higher compared to the proportion fortified foods falling within the Wheel of Five. Examples of non-fortified foods consumed within the DNFCS 2012–2016 and falling within the Wheel of Five included whole-grain products, brown rice, vegetable oils and milk, yoghurt and low-fat cheeses. Consumed fortified foods falling within the Wheel of Five contained whole-grain breads, white breads enriched with fibre and micronutrients, margarines, soy milk and yoghurt (drinks). Non-fortified foods falling outside the Wheel of Five included all sorts of white wheat products, including bread, pasta and wraps, other grains, including couscous and white rice, butter and high-fat milk and -cheeses and custard. Examples of fortified foods consumed and falling outside the Wheel of Five were breakfast cereals, fats used for frying, hard margarines, soy drinks, milk drinks and quark.

**Table 3:** Percentage of fortified and non-fortified foods within the Wheel of Five consumed within the DNFCS 2012–2016

Food group	Consumed fortified foods		Consumed non-fortified foods	
	Within Wheel of Five (%)	Outside Wheel of Five (%)	Within wheel of Five (%)	Outside Wheel of Five (%)
Dairy products and substitutes	36	64	38	62
Cereals and cereal products	22	78	40	60
Meat, meat products and substitutes	0	100	21	79
Fats and oils	60	40	53	47
Sugar and confectionery	0	100	0	100
Cakes and sweet biscuits	0	100	0	100
Non-alcoholic beverages	0	100	71	29
Condiments, spices, sauces and yeast	5	95	3	97
Miscellaneous	0	100	1	99

### Contribution of fortification to the total intake of micronutrients of users fortified foods

**Fig. 2:**
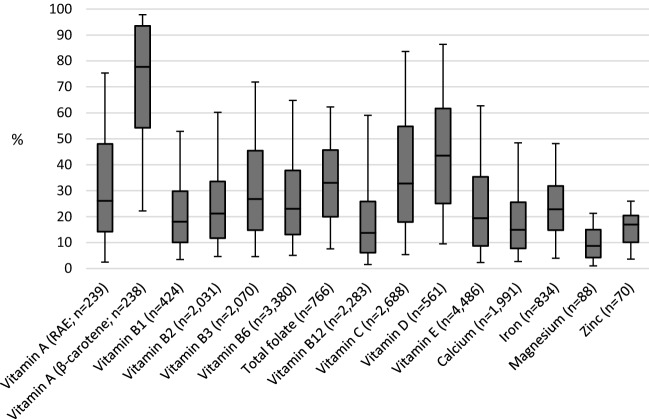
Contribution of fortification to the total micronutrient intake of users of fortified products fortified with these specific nutrients. Upper whisk: P95, upper part boxplot: P75, middle line boxplot: P50, lower part boxplot: P25, lower whisk: P5, *n* amount of recall days on which a food fortified with that specific nutrient was consumed. Data only shown for micronutrients for which there was consumption on more than 6 recall days

Among fortified food users the median contribution to the total micronutrient intake was the highest for vitamin A (β-carotene), D, C and total folate with, respectively, a median contribution of 78%, 44%, 33% and 33% (Fig. 2). For all other micronutrients, median contribution to total intake was between 9 and 30%. Foods fortified with vitamin E and B6 were most frequently consumed; on between 39 and 52% of the recall days. Foods fortified with β-carotenes were only consumed on 3% of all recall days. See Online Appendix 2 for a total overview of all voluntary fortified foods consumed within the DNFCS 2012–2016 for each micronutrient.

### Habitual micronutrient intake of (non-)users of fortified foods

**Fig. 3:**
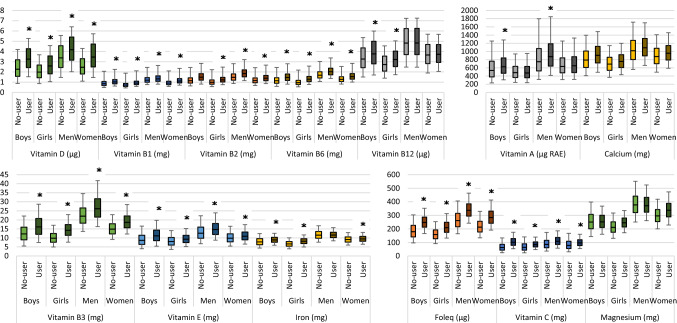
Habitual micronutrient distributions for users and non-users. Lower whisk: P5, bottom boxplot: P25, line in middle: P50, top boxplot: P75, upper whisk: P95, *significant higher median habitual intake among users (not possible for magnesium)

Median habitual micronutrient intakes of users of fortified foods were significantly higher compared to non-users for vitamin B1, B2, B3, B6, C, D and E and folate equivalents for all ages, for vitamin B12 and iron amongst children, for vitamin A (RAE) among boys and men and for calcium among boys and adults (Fig. 3). The median habitual intakes were 4% (for iron) to 64% (for vitamin C) higher among users compared to non-users.

Table 4 shows the higher median habitual intakes resulted in a lower proportion of adult users with intakes below the EAR for vitamins A (boys and men), B1 (men), B2, B6, and C and folate equivalents, calcium and iron (men). For children aged 14–17 years, the proportion children with intakes below the EAR for vitamin C and E and iron was lower among users.

**Table 4:** The assessment of inadequacy of micronutrient intakes separate for users and non-users of fortified fats

Micronutrient	Age (year)	Gender	AI	EAR	Non-users		Users	
					Evaluation risk inadequate intake for each age (in years)	% < EAR	Evaluation risk inadequate intake	% < EAR^a^
**Vitamin A** **(µg RAE)**	1–17	Boys	300/350/400/ 600^b^	600^c^	1–9: LR 10–13: NSP	55.7 (48.5–59.7)	1–9, 12–13: LR 10–11: NSP	**40.9 (35.1–46.5)**
		Girls		500^c^	1–9: LR 10–13: NSP	48.4 (42.2–56.7)	1–9: LR 10–13: NSP	50.1 (44.5–55.1)
	18–79	Men	–	615	–	31.9 (27.0–36.6)	–	26.8 (22.9–30.6)
		Women		525	–	25.5 (18.4–34.4)	–	34.4 (30.9–38.6)
**Vitamin B1 (mg)**	1–17	Boys	0.3/0.5/0.8/1.1	–	1–13: LR 14–17: NSP	–	1–13, 15–17: LR 14: NSP	–
		Girls			1–13: LR 14–17: NSP	–	1–13: LR 14–17: NSP	–
	18–79	Men	–	0.072	–	1.2 (1.2–1.2)	–	**1.3 (1.3–1.4)**
		Women			–	0.9 (0.9–0.9)	–	**1.1 (1.1–1.2)**
**Vitamin B2 (mg)**	1–17	Boys	0.5/0.7/1/1.5	–	1–13: LR 14–17: NSP	–	LR	–
		Girls	0.5/0.7/1/1.1		1–8, 10–11, 13: LR 9, 12, 14–17: NSP	–	LR	–
	18–79	Men	–	1.3	–	33.4 (30.0–38.8)	–	**10.6 (6.9–13.9)**
		Women			–	64.3 (61.1–67.5)	–	**40.0 (34.8–45.2)**
**Vitamin B3 (mg)**	1–17	Boys	4/7/11/17	–	1–13, 17: LR 14–16: NSP	–	1–13, 15– 17: LR 14: NSP	–
		Girls	4/7/11/13		1–8, 12–13: LR 9–11, 14–17: NSP	–	1–13, 15–17: LR 14: NSP	–
	18–79	Men	–	1.3	–	0 (0–0)	–	0 (0–0)
		Women			–	0 (0–0)	–	0 (0–0)
**Vitamin B6 (mg)**	1–17	Boys	0.4/0.7/1.1/1.5	–	1–13: LR 14–17: NSP	–	LR	–
		Girls			1–8: LR 9–17: NSP	–	1–13: LR 14–17: NSP	–
	18–79	Men	–	1.1	–	11.6 (7.3–17.8)	–	5.8 (5.1–8.6)
		Women			–	26.2 (21.1–35.1)	–	20.3 (16.9–24.1)
**Folate equivalents (µg)**	1–17	Boys	85/150/225/300	–	1–3, 6–8: LR 4–5, 9–17: NSP	–	1–13: LR 14–17: NSP	–
		Girls			1–3, 6–8: LR 4–5, 9–17: NSP	–	1–8: LR 9–17: NSP	–
	18–79	Men	–	200	–	17 (14.3–19.8)	–	**0.5 (0.0–1.2)**
		Women			–	40.5 (37.2–44.6)	–	**7.3 (3.6–13.1)**
**Vitamin B12 (µg)**	1–17	Boys	0.7/1.3/2/2.8	–	LR	–	LR	–
		Girls			1–13, 16–17: LR 14–15: NSP	–	LR	–
	18–79	Men	–	2	–	1.6 (0.9–2.5)	–	1.6 (0.3–4.0)
		Women			–	6.4 (4.8–8.6)	–	4.3 (2.1–6.5)
**Vitamin C (mg)**	1–17	Boys	25/30/40/50^b^	60^c^	LR	39.2 (35.2–46.2)	LR	**8.0 (5.1–10.7)**
		Girls		50^c^	LR	29.9 (26.6–35.2)	LR	**6.5 (3.5–8.8)**
	18–79	Men	–	60	–	23.8 (21.7–28.8)	–	**6.1 (3.8–8.4)**
		Women		50	–	20.2 (17.2–23.6)	–	**3.0 (1.6–4.6)**
**Vitamin D (µg)**	1–17	Boys	10/3^d^	–	NSP/ 1: LR 2–17: NSP^5^	–	NSP/ LR^5^	–
		Girls			NSP/ NSP^5^	–	NSP/ NSP^5^	–
	18–79	Men	10/3^d^	10	NSP/ 26–69:LR 18–25: NSP^5^	99.4 (98.5–99.9)	NSP/ LR^5^	98.6 (96–100.5)
		Women			NSP/ NSP^5^	100 (99.9–100.0)	NSP/ 18, 26–27, 31–69: LR 19–25, 28–30: NSP^5^	99.2 (96.4–100.2)
**Vitamin E (mg)**	1–17	Boys	4/5/6/8^b^	6^c^	LR	5.5 (2.5–8.4)	LR	**0.6 (0.6–1.1)**
		Girls	4/5/6/7^b^	5^c^	LR	6 (3.0–8.0)	LR	**1.3 (1.3–2.1)**
	18–79	Men	13	–	25–58: LR 18–24, 59–79: NSP	–	18–72, 74: LR 73, 75–79: NSP	–
		Women	11		NSP	–	37, 39–41, 43–45, 47–48, 50–51, 54–78: LR 18–36, 38, 42, 46, 49, 52–53, 79: NSP	–
**Iron (mg)**	1–17	Boys	8/9/11^b^	7^c^	NSP	12.5 (9.3–15.2)	4–9: LR 1–3, 10–13: NSP	**2.9 (0.0–5.2)**
		Girls		10^c^	NSP	89.4 (86.6–92.0)	5: LR 1–4, 6–13: NSP	**71.0 (65.6–77.9)**
	18–79	Men	–	6	–	0.6 (0.3–0.9)	–	**0.1 (0.0–0.2)**
		Women		7/6	–	9.4 (9.3–13.3)	–	7.2 (2.9–11.2)
**Calcium (mg)**	1–17	Boys	500/700/1200	–	1–8: LR 9–17: NSP	–	1–8: LR 9–17: NSP	
		Girls	500/700/1100		1–3: LR 4–17: NSP	–	1–3, 5–8: LR 4, 9–17: NSP	
	18–79	Men	1200^e^	860/ 750^f^	NSP	18–24: 41.0 (40.2–45.3) 25–69: 17.9 (18.1–23.3)^g^	NSP	18–24: 27.1 (29.3–37.0)^h^ 25–69: 11.0 (10.1–16.0)
		Women	1100/1200^e^		NSP	18–24: 64.4 (61.2–67.6) 25–49: 32.4 (29.6–42.9)	NSP	18–24: 48.7 (44.2–56.1) 25–49: 22.0 (16.1–28.0)

*LR* low risk, *NSP* no statement possible

^a^Statistical significant lower proportion of users below the EAR compared to non-users is indicated when valued are displayed bold

^b^The AI accounts only for children 1–13 years old

^c^The EAR for children accounts only for children 14–17 years old

^d^Two AI-values for vitamin D, where 3 µg/day indicates adequate vitamin D intake with enough sun exposure and 10 µg/day if this amount of sun exposure is not met

^e^AI for men 70–79 and women 50–79 years old

^f^EAR = 860 for adults aged 18–24 year. EAR = 750 for women 25–50 year old and men 25–70 year old

^g^Estimation falls outside of 95%CI due to extreme values (n=5; 0.5% of all observations within this subgroup)

^h^Estimation falls outside of 95%CI due to an extreme value (n=1; 1.5% of all observations within this subgroup)

Among users of voluntary fortified foods, more age groups have a low risk on inadequate vitamin A (boys), B1 (boys), B2, B3, B6, folate equivalents, vitamin B12 (girls), D (assuming enough sunlight exposure), E, calcium (girls) and iron intake (Table 4). Non-user children for vitamin C and non-user boys for vitamin B12 already had a low risk on inadequate intakes, therefore, the higher intakes among users did not change the assessment of the risk on inadequate intakes.

Although higher intakes were observed among users for vitamin B6, D and E and calcium, this did not lead to a significant higher proportion of users exceeding the UL, compared to non-users (Online Appendix 3).

## Discussion

Based on a 2-day recall, three-quarters of the Dutch population can be considered as a user of voluntary fortified foods. Generally, one type of fortified food a day was consumed. Fats and oils, non-alcoholic beverages and dairy products were food groups from which fortified food consumption mostly was reported. Most of the consumed fortified foods do not fall within the Wheel of Five, implicating that these foods are no healthy choice. Habitual intakes of users of voluntary fortified foods were higher for vitamins A, B1, B2, B3, B6, B12, C, D and E, calcium, folate equivalents and iron, compared to non-users, resulting into a lower proportion with inadequate intakes for vitamin A, B1, B2, B6, C, calcium, iron and folate equivalents among (male) adults.

The present study showed the consumed voluntary fortified foods within the Dutch population were most frequently from the food groups ‘fats and oils’, ‘non-alcoholic beverages’ and ‘dairy products’. This is somewhat comparable to the consumption in other countries. Comparable to the Netherlands, these were also main voluntary fortified food groups in e.g. Finland (yoghurt), Poland (non-alcoholic beverages and dairy), USA (milk and milk drinks) and Ireland (fat spreads) [21–24]. Due to different food habits, in Finland, Ireland, Poland and USA ready-to-eat-breakfast cereals is an important voluntary fortified food group, in contrast to the Netherlands [21–24]. In Ireland, 53% of the population consumes ready-to-eat-breakfast cereals, in the Netherlands, this is only on 3% of the recall days. In Finland, Poland and the USA, fortified fruit juices are often consumed fortified foods, while in the Netherlands, these fortified juices are hardly consumed (1% of the recall days). Other fortified non-alcoholic beverages, including carbonated, soft- and isotonic drinks, are consumed on a larger scale in the Netherlands (on 19% of all recall days).

The current study showed voluntary fortified foods contributed to micronutrient intakes and habitual vitamin A, B1, B2, B3, B6, B12, C, D and E, calcium, iron and folate-equivalent-intakes were higher among users, compared to non-users. Although fortified foods have a higher micronutrient content, the current study showed they mostly cannot be classified as a healthy food, according to the definitions of the Wheel of Five. Therefore, the current study suggests that a food pattern consisting of voluntary fortified foods results into higher micronutrient intakes, but may also contribute to higher fat, energy, sugar, salt or lower fibre intake. Hennesy et al. found the mean (saturated) fat contribution of consumed voluntary fortified foods in Ireland to mean daily (saturated) fat intake was below the contribution of voluntary fortified foods to energy intake [22]. They, therefore, assumed consumption of voluntary fortified foods does not have an impact on high (saturated) fat intakes. On the other hand, mean total sugar and dietary fibre contributions were comparable to mean energy contribution. As fortified foods differ among the Irish and Dutch populations, contributions to macronutrient intake may also vary. Additional research could study the contribution of voluntary fortified foods to the total fat, energy, sugar, salt and fibre intake, to see if the suggestion that consuming voluntary fortified foods, generally not included in the Wheel of Five, are indeed resulting in unfavourable intakes of these compounds.

Although voluntary food fortification is allowed in the Netherlands, it is not promoted by the Dutch government to improve micronutrient intakes via voluntary fortified foods. Exception to this is the addition of vitamin A and D to fats and iodine to bakery salt, which is encouraged by covenants. However, these were not included in this study for that reason. Legislation for voluntary fortification is only intended to prevent excessive intakes in the whole population. The current study showed higher habitual intakes were observed for most of the nutrients studied, among users of fortified foods. However, among users and non-users of voluntary fortified foods, the intake remained below UL for all micronutrients with an UL. From this, we can conclude that the Dutch policy on voluntary fortification successfully prohibits excessive intakes from food sources only. However, intake from dietary supplements was not included in our study.

Van Rossum et al. showed, in the total Dutch adult population, risks on low intakes exist for vitamin A, B2, B6 (women), C, total folate, vitamin D (70 +) and calcium (19–70 for men and 19–50 for women), based on the intake of food and supplements [6]. The current study showed habitual vitamin A, B1, B2, B6, C, calcium and total folate-equivalent intakes were higher among users, which contributed to a lower proportion below the EAR for those nutrients. Except for vitamin A, B2 and calcium, this resulted in a proportion below the EAR < 10% among users of foods fortified with these specific micronutrients, although among non-users these proportions were > 10%. Also in other countries, voluntary food fortification contributed to a lower risk on inadequate intakes. Comparing the Irish population with and without inclusion of their added micronutrient intake, lower proportions below the EAR were observed for vitamin D, folate (women) and iron (women) [22]. Among Northern-Irish women in the childbearing age voluntary food fortification with folic acid resulted into an increase in women with a favourable red cell folate status, which is associated with a prevention of neural tube defects [25]. In the US, a 2–95% increase in proportion below the EAR for calcium, folate, iron, magnesium, vitamin A, B2, B3, B6, B12, C, D, E and zinc was calculated when food fortification of ready-to-eat breakfast cereals were not included, compared to when it was included [26]. This shows consumption of fortified foods can have an impact on lowering risks on inadequate intakes at a population level. However, this requires an intake in a substantial part of the population. With the current practice of voluntary fortification, only a part of specific food groups are fortified with some micronutrients. In addition, the part of the population consuming those fortified foods is limited. For B1 and folate-equivalents, this was only on less than 10% of the observed recall days. Even though, among consumers of these foods, the impact may be big.

Besides the allowance of voluntary food fortification, enrichment of fats with vitamin A and D is encouraged in the Netherlands. de Jong et al. [27] showed users of these fats generally had significantly higher vitamin A and D intakes compared to non-users. In the current study, users of these types of fats were only included as user if the consumed fat was also fortified with other nutrients, or the user consumed another type of voluntary fortified food. As significantly more users of voluntary foods used fortified fats compared to non-users, there is a possibility the difference in vitamin D-intakes among all users and non-users of voluntary fortified foods and the vitamin A-intakes among male users and non-users are smaller than calculated in the current study. It was not possible to calculate the habitual intakes of users and non-users of voluntary fortified foods who did not use fortified fats, as groups became too small to calculate habitual intakes (91% of the users used fortified fats). In a future study, habitual vitamin A and D intakes can be calculated excluding the intake from fortified fats, using for example scenario calculations.

In the current study, the intake of micronutrients added to foods was assumed to be equal to the amount of nutrients declared on the package. This is comparable to methods from other studies, including the Irish study by Hennessy et al*.* [22]. Using this method, there is a possibility of over- as well as underestimating the (habitual) intake of micronutrients added to foods. A study measuring the chemical composition of foods and supplements showed that the measured value could over- as well as underestimate the labelled value [28]. It is not possible to predict this. The contribution of fortified food to the total intake did include the micronutrient value that is naturally in fortified foods as well as the added amounts. In general, it is assumed that the added amounts are much larger than the natural value. Using vitamin C as an example, only a few non-fortified alternatives for foods fortified with vitamin C naturally contained vitamin C (vegetable- and fruit juices, breakfast fruit drinks, ice cream, chocolate (milk) and yoghurt), and vitamin C content of the alternatives was only high for fruit- and/or vegetable juices (between 8 and 12 mg/100 g product compared to 9–27 mg/100 fortified product). Based on this, we do not expect a high overestimation due to this assumption. On the other hand, declarations on food labels may underestimate true nutrient content of fortified foods. To assure the nutrient content during the total shelf life, a higher content of micronutrients is added to compensate for possible expiration of these nutrients over time. An American study showed five of the six investigated cereals contained higher vitamin D than declared on the package, variating from 105 to 228% [29]. Underestimating would resulted into even higher contributions from fortification to total micronutrient intake and also higher habitual intakes among users. In the current study, users had a low risk on excessive intakes, however, this risk may be higher if the true micronutrient was known. Future studies may estimate the under- or overestimating of the contribution and habitual intakes using only analysed micronutrient content, rather than the declarations of food labels.

Micronutrients can be consumed via foods and drinks or dietary supplements. The aim of our study was to study the effect of food fortification on the micronutrient intake from foods, therefore, the intake from dietary supplements was not included. The questions raises if conclusions on inadequacy is different when intake from food supplements was considered. Within the DNFCS 2012–2016, the percentage of self-reported supplement users was 42% [6], and these users were equally distributed over users and non-users of voluntary fortified foods (data not shown). On average, among children, food supplements contributed to 6% of the total median habitual intake of vitamins and to 4% of the minerals. For adults, these contributions were, respectively, about 10% for vitamins and 5% for minerals. Exception is vitamin D with a high contribution among children (18%). This is due to the supplementation advice for young children (− 4 years). Van Rossum et al. calculated micronutrient intakes from both foods as from foods and supplements together [6]. Including supplement intake to the results of this study might only change the conclusion for iron intake from low intakes to adequate. Since contribution of food supplements is low, we assume conclusions on inadequate intakes for the other nutrients will not change in the current study.

The current study was based on data from the DNFCS 2012–2016. This representative study for the Dutch population provided the current study with high-quality data on the population characteristics and their intakes, using an extensive questionnaire and elaborate food consumption information linked to the NEVO-database. This comprehensive database includes most of the foods consumed within the Dutch population. For most of the products within DNFCS 2012–2016, the micronutrient content is known, as the coverage of the micronutrients reported in current study within this database is high, ranging from 71 to 99%. For those foods of which micronutrient content was not known, we assumed micronutrient content was equal to zero. This may underestimated true content. NEVO is a database focussing on creating nutrient values for generic foods. Since fortified foods often differ among manufactures, only generic foods are created when the composition of different fortified foods are really equal. If not, separate codes with each its own nutrient composition are created. The database is carefully created and updated every 2 years. However, the composition of fortified foods may change, and these changes might not be covered in the database version used for current study. Intake from fortified foods can therefore be over- or underestimated. Also, as intakes may vary from day to day, it was not possible to make conclusions solely on the intake on 2 recall days. We, therefore, used SPADE to correct for those within-person variances, resulting in habitual intakes for the total population. Conclusions could be made, based on usual intakes, rather than daily intakes and these habitual intakes could be compared to dietary reference values.

## Conclusion

Current study showed micronutrients voluntary added to foods increased micronutrient intakes, decreased the risk on inadequate micronutrient intakes for some micronutrients and did not increase the risk on excessive intakes. However, most voluntary fortified foods cannot be considered as healthy foods, therefore, consumption of these foods is not the preferred first choice to increase micronutrient intake. Continuing monitoring micronutrient intakes and the role of voluntary foods is important to keep an eye on adequate and excessive intakes. In future studies, the association between the higher micronutrient intakes and (potential) excessive intake of e.g. saturated fat, sugar and salt should be studied. This is important to better understand the role of voluntary fortified foods in increasing micronutrient intake in a healthy food pattern.

## Supplementary Information

Below is the link to the electronic supplementary material.

Supplementary file1 (DOCX 15 kb)

Supplementary file2 (DOCX 39 kb)

Supplementary file3 (DOCX 27 kb)

## Data Availability

Data of the DNFCS 2012–2016 are available on request from https://www.rivm.nl/en/dutch-national-food-consumption-survey/data-on-request (accessed on 20 May 2021).
