# Supplementary material for: Contribution of voluntary fortified foods to micronutrient intake in The Netherlands
Source: Eur J Nutr. 2022 Jan 1;61(3):1649–63. doi: 10.1007/s00394-021-02728-4 (PMC8921121; doi:10.1007/s00394-021-02728-4)
Supplement: Supplementary file 1 — Supplementary file1 (DOCX 15 kb) [file 394_2021_2728_MOESM1_ESM.docx]

**Appendix 1 – Wheel of Five categories**

Foods falling within the Wheel of Five for the categories relevant for fortification [26]

|  | Within the Wheel of Five | Not within the Wheel of Five |
| --- | --- | --- |
| Dairy products and substitutes | Skimmed and semi-skimmed (butter-) milk, | Dairy- and soydrinks with too much sugar ≥6 g sugar/100 g drinks |
|  | Skimmed and semi-skimmed yoghurt | Pudding and desserts with added sugar |
|  | Skimmed quark | Full-fat milk and full-fat yoghurt |
|  | Drinking yoghurt | Rice- and almond drinks |
|  | Soy drinks with added vitamin B12 and calcium | Custard |
|  | 10+, 20+ or 30+ cheese with ≤2 g salt /100 g cheese | (yoghurt-)Ice cream (milk based) |
|  | Dairy spread | 48+ and 60+ cheese |
|  | Cottage cheese | Feta |
|  | Mozzarella | Crème fraiche, sour cream, cooking cream |
|  | Fresh goat cheese | Whipped cream |
| Cereals and cereal products | Whole wheat bread and -rolls | White bread |
|  | Brown bread and -rolls | White crispbread |
|  | Whole wheat crispbread | Rusk |
|  | Whole wheat raisinbread and -mueslibread | Raisinbread |
|  | Ryebread | Croissants |
|  | Oatmeal, muesli and other whole wheat cereals with ≤16 g sugar/100 g and no added salt | Cereals with > 16 g sugar/100 g |
|  | Whole wheat pasta | White pasta |
|  | Whole wheat mie and -noodles | White mie and noodles |
|  | Brown rice | White rice |
|  | Whole wheat bulgur | White couscous |
|  | Couscous with ≥ 2.1 g fibre/100 g |  |
|  | quinoa |  |
| Meat, meat products and substitutes | Unprocessed meat | All cold cuts |
|  | Ready-to-eat vegetarian products with salt ≤ 1.1g/100 g | Processed meat |
|  |  | Fatty meat |
|  |  | Ready to eat vegetarian products with salt >1.1 g/100 g |
| Fats and oils | Soft margarines used for bread | Hard margarines |
|  | Soft margarines and cooking fats | Hard cooking fats |
|  | Most vegetable oils | Hard frying fats |
|  |  | Butter |
|  |  | Ghee |
|  | Cocos oil |  |
|  | Palm oil |  |
| Sugar and confectionery | None | All |
| Cakes and sweet biscuits | None | All |
| Non-alcoholic beverages | Water | Beverages with sugar |
|  | Black- and green tea | Light-soda |
|  | Herbs tea (limited due toxicity or blood pressure increasing effects of certain herbs) | Alcohol beverages |
|  | Coffee (limited due to caffeine and cafestol content) | Cooking coffee and coffee from a cafetière |
| Condiments, spices, sauces and yeast | None | All |
