# Supplementary material for: Contribution of voluntary fortified foods to micronutrient intake in The Netherlands
Source: Eur J Nutr. 2022 Jan 1;61(3):1649–63. doi: 10.1007/s00394-021-02728-4 (PMC8921121; doi:10.1007/s00394-021-02728-4)
Supplement: Supplementary file 2 — Supplementary file2 (DOCX 39 kb) [file 394_2021_2728_MOESM2_ESM.docx]

Appendix 2 – Overview to what foods micronutrients are added to

The frequency shows how often the food was consumed within the DNFCS 2012-2016. The percent shows the contribution of that food to the total intake of voluntary fortified foods within that micronutrient category.

### Vitamin A (RAE)

| **Name** | **Frequency** | **Percent** |
| --- | --- | --- |
| Drank ACE drink | 22 | 7,07 |
| Drank Taksi/Djoezz en soortgelijke | 16 | 5,14 |
| Drank vruchten- 1 vrucht (geen 100% sap) | 10 | 3,22 |
| Drank vruchten- gemengd (geen 100% sap) | 193 | 62,06 |
| Dressing olie-azijn | 2 | 0,64 |
| Dressing sla- naturel zonder vet | 2 | 0,64 |
| Dressing, n.s. | 2 | 0,64 |
| Frisdrank Dubbelfrisss en soortgelijke | 4 | 1,29 |
| Frisdrank met vruchtensap | 21 | 6,75 |
| Ovomaltine (cacaopoeder) | 1 | 0,32 |
| Sap vruchten en/of groente- concentraat | 1 | 0,32 |
| Sap vruchten- 1 vrucht (100% sap) | 3 | 0,96 |
| Sap vruchten- gemengd (100% sap) | 28 | 9,00 |
| Sap vruchten/groente- | 1 | 0,32 |
| Sap/drank vruchten-, n.s. | 3 | 0,96 |
| Slasaus | 2 | 0,64 |

### Vitamin A (β-carotene)

| **Name** | Frequency | Percent |
| --- | --- | --- |
| Drank ACE drink | 22 | 6,83 |
| Drank Taksi/Djoezz en soortgelijke | 16 | 4,97 |
| Drank vruchten- 1 vrucht (geen 100% sap) | 10 | 3,11 |
| Drank vruchten- gemengd (geen 100% sap) | 205 | 63,66 |
| Dressing olie-azijn | 2 | 0,62 |
| Dressing sla- naturel zonder vet | 2 | 0,62 |
| Dressing, n.s. | 2 | 0,62 |
| Frisdrank Dubbelfrisss en soortgelijke | 4 | 1,24 |
| Frisdrank met vruchtensap | 21 | 6,52 |
| Sap vruchten en/of groente- concentraat | 1 | 0,31 |
| Sap vruchten- 1 vrucht (100% sap) | 3 | 0,93 |
| Sap vruchten- gemengd (100% sap) | 28 | 8,70 |
| Sap vruchten/groente- | 1 | 0,31 |
| Sap/drank vruchten-, n.s. | 3 | 0,93 |
| Slasaus | 2 | 0,62 |

### Vitamin B1

| **Name** | Frequency | Percent |
| --- | --- | --- |
| Biscuit meergranen- | 1 | 0,28 |
| Biscuit volkoren- | 1 | 0,28 |
| Biscuit/koekje voor ontbijt | 10 | 2,76 |
| Brood bruin/tarwe verrijkt (vezel/vitamines e.d.) | 37 | 10,22 |
| Brood meergranen- verrijkt (vezel/vitamines e.d.) | 2 | 0,55 |
| Brood wit verrijkt (vezel/vitamines e.d.) | 68 | 18,78 |
| Brood, verrijkt (vezel/vitamines e.d.), n.s. | 2 | 0,55 |
| Cacaopoeder | 49 | 13,54 |
| Cornflakes | 27 | 7,46 |
| Cracker luchtige gewone (wit) | 1 | 0,28 |
| Cracker luchtige volkoren- | 6 | 1,66 |
| Cracker luchtige, n.s. | 5 | 1,38 |
| Cracker overige volkoren | 1 | 0,28 |
| Drinkontbijt op fruitbasis | 2 | 0,55 |
| Koek muesli- | 1 | 0,28 |
| Koekje gewoon zonder creme/glazuur | 1 | 0,28 |
| Koekje kinder- | 1 | 0,28 |
| Koekje, n.s. | 1 | 0,28 |
| Liga en soortgelijke biscuit continue | 1 | 0,28 |
| Melk chocolade- gewone | 20 | 5,52 |
| Muesli krokante | 1 | 0,28 |
| Muesli ongeroosterd | 1 | 0,28 |
| Mueslireep gewoon | 2 | 0,55 |
| Ontbijtgraan flakes andere dan cornflakes, n.s. | 4 | 1,10 |
| Ontbijtgraan koekvorm Weetabix en soortgelijke | 4 | 1,10 |
| Ontbijtgraan krispies/pops | 5 | 1,38 |
| Ontbijtgraan loops/rondjes | 17 | 4,70 |
| Ontbijtgraan voor pap | 59 | 16,30 |
| Ontbijtgraan voor pap kinder- | 14 | 3,87 |
| Ontbijtgraan, n.s. | 1 | 0,28 |
| Rijstwafel zoete | 16 | 4,42 |
| Time Out granenbiscuit en soortgelijke | 1 | 0,28 |

### Vitamin B2

| **Name** | Frequency | Percent |
| --- | --- | --- |
| Amandeldrank Alpro en soortgelijke | 28 | 2,44 |
| Brood meergranen- verrijkt (vezel/vitamines e.d.) | 7 | 0,61 |
| Brood, n.s. | 1 | 0,09 |
| Cornflakes | 145 | 12,64 |
| Cracker luchtige gewone (wit) | 1 | 0,09 |
| Cracker luchtige volkoren- | 6 | 0,52 |
| Cracker luchtige, n.s. | 5 | 0,44 |
| Cracker overige volkoren | 1 | 0,09 |
| Drank energie- (met cafeine) | 88 | 7,67 |
| Drank vruchten- 1 vrucht (geen 100% sap) | 8 | 0,70 |
| Drank vruchten- gemengd (geen 100% sap) | 86 | 7,50 |
| Drinkontbijt op yoghurtbasis | 13 | 1,13 |
| Drinkontbijt, n.s. | 2 | 0,17 |
| Frisdrank Dubbelfrisss en soortgelijke | 4 | 0,35 |
| Frisdrank met vruchtensap | 21 | 1,83 |
| Frisdrank, n.s. | 2 | 0,17 |
| Gistextract Marmite en soortgelijke | 19 | 1,66 |
| Haverdrank Alpro en soortgelijke | 2 | 0,17 |
| Koek ontbijt- gevuld of met smaakje | 2 | 0,17 |
| Koek ontbijt- volkoren ongevuld | 1 | 0,09 |
| Koekje kinder- | 1 | 0,09 |
| Kwark gewone | 29 | 2,53 |
| Kwark, n.s. | 1 | 0,09 |
| Kwarkyoghurt | 5 | 0,44 |
| Melk, n.s. | 2 | 0,17 |
| Melkdrank geen chocolade/anijs | 56 | 4,88 |
| Muesli krokante | 6 | 0,52 |
| Muesli ongeroosterd | 2 | 0,17 |
| Muesli, n.s. | 1 | 0,09 |
| Mueslireep gewoon | 6 | 0,52 |
| Mueslireep met yoghurtlaagje | 1 | 0,09 |
| Ontbijtgraan flakes andere dan cornflakes, n.s. | 29 | 2,53 |
| Ontbijtgraan koekvorm Weetabix en soortgelijke | 4 | 0,35 |
| Ontbijtgraan krispies/pops | 48 | 4,18 |
| Ontbijtgraan kussentje | 12 | 1,05 |
| Ontbijtgraan loops/rondjes | 41 | 3,57 |
| Ontbijtgraan voor pap | 7 | 0,61 |
| Ontbijtgraan, n.s. | 5 | 0,44 |
| Ovomaltine (cacaopoeder) | 1 | 0,09 |
| Pudding yoghurt- (niet luchtig) | 1 | 0,09 |
| Rijstdrank Ricedream en soortgelijke | 4 | 0,35 |
| Sap vruchten en/of groente- concentraat | 1 | 0,09 |
| Sap vruchten- 1 vrucht (100% sap) | 3 | 0,26 |
| Sap vruchten- gemengd (100% sap) | 30 | 2,62 |
| Sap/drank vruchten-, n.s. | 2 | 0,17 |
| Sojadessert | 16 | 1,39 |
| Sojadrink/melk | 215 | 18,74 |
| Sojavla | 15 | 1,31 |
| Sojayoghurt | 24 | 2,09 |
| Vla gewone | 32 | 2,79 |
| Vla, n.s. | 1 | 0,09 |
| Yoghurt Griekse (ingedikte yoghurt) | 7 | 0,61 |
| Yoghurt drink- | 34 | 2,96 |
| Yoghurt gewone (koe) | 60 | 5,23 |
| Yoghurt, n.s. | 2 | 0,17 |
| ZZZ-OUD NIET GEBRUIKEN Yoghurt & Vla kant-en-klaar | 1 | 0,09 |

### Vitamin B3

| **Name** | Frequency | Percent |
| --- | --- | --- |
| Brood bruin/tarwe verrijkt (vezel/vitamines e.d.) | 37 | 0,99 |
| Brood meergranen- verrijkt (vezel/vitamines e.d.) | 2 | 0,05 |
| Brood wit verrijkt (vezel/vitamines e.d.) | 63 | 1,68 |
| Brood, verrijkt (vezel/vitamines e.d.), n.s. | 2 | 0,05 |
| Cacaopoeder | 49 | 1,30 |
| Cornflakes | 129 | 3,44 |
| Cracker luchtige gewone (wit) | 1 | 0,03 |
| Cracker luchtige volkoren- | 6 | 0,16 |
| Cracker luchtige, n.s. | 5 | 0,13 |
| Cracker overige volkoren | 1 | 0,03 |
| Diksap onverdund | 1 | 0,03 |
| Drank ACE drink | 3 | 0,08 |
| Drank Taksi/Djoezz en soortgelijke | 1 | 0,03 |
| Drank energie- (met cafeine) | 169 | 4,50 |
| Drank sport- energy | 3 | 0,08 |
| Drank sport- isotone | 10 | 0,27 |
| Drank sport-, n.s. | 6 | 0,16 |
| Drank vruchten- 1 vrucht (geen 100% sap) | 8 | 0,21 |
| Drank vruchten- gemengd (geen 100% sap) | 283 | 7,54 |
| Drinkontbijt op fruitbasis | 2 | 0,05 |
| Frisdrank Dubbelfrisss en soortgelijke | 7 | 0,19 |
| Frisdrank met vruchtensap | 46 | 1,23 |
| Frisdrank sinas | 6 | 0,16 |
| Frisdrank vruchtensmaak Crystal Clear en soortgelijke | 10 | 0,27 |
| Frisdrank, n.s. | 5 | 0,13 |
| Gistextract Marmite en soortgelijke | 19 | 0,51 |
| Koek ontbijt- gevuld of met smaakje | 2 | 0,05 |
| Koek ontbijt- volkoren ongevuld | 1 | 0,03 |
| Koekje kinder- | 1 | 0,03 |
| Melk chocolade- gewone | 20 | 0,53 |
| Muesli krokante | 6 | 0,16 |
| Muesli ongeroosterd | 2 | 0,05 |
| Muesli, n.s. | 1 | 0,03 |
| Mueslireep gewoon | 6 | 0,16 |
| Mueslireep met yoghurtlaagje | 1 | 0,03 |
| Ontbijtgraan flakes andere dan cornflakes, n.s. | 27 | 0,72 |
| Ontbijtgraan koekvorm Weetabix en soortgelijke | 4 | 0,11 |
| Ontbijtgraan krispies/pops | 42 | 1,12 |
| Ontbijtgraan kussentje | 1 | 0,03 |
| Ontbijtgraan loops/rondjes | 22 | 0,59 |
| Ontbijtgraan voor pap | 2 | 0,05 |
| Ontbijtgraan, n.s. | 5 | 0,13 |
| Ovomaltine (cacaopoeder) | 1 | 0,03 |
| Sap vruchten en/of groente- concentraat | 1 | 0,03 |
| Sap vruchten- 1 vrucht (100% sap) | 3 | 0,08 |
| Sap vruchten- gemengd (100% sap) | 46 | 1,23 |
| Sap/drank vruchten-, n.s. | 3 | 0,08 |
| Siroop limonade- gewone (vruchtenbasis) | 1650 | 43,94 |
| Siroop limonade- gewone (vruchtenbasis) verdund verhouding onbekend | 755 | 20,11 |
| Siroop limonade- rozenbottel speciale | 27 | 0,72 |
| Siroop limonade- rozenbottel speciale verdund verhouding onbekend | 1 | 0,03 |
| Siroop limonade- rozenbottel vruchtenmix | 39 | 1,04 |
| Siroop limonade- rozenbottel vruchtenmix verdund | 12 | 0,32 |
| Siroop limonade- rozenbottel, n.s. | 33 | 0,88 |
| Siroop limonade-, n.s. | 33 | 0,88 |
| Siroop limonade-, n.s. verdund verhouding onbekend | 82 | 2,18 |
| Water vitamine- en soortgelijke | 52 | 1,38 |

### Vitamin B6

| **Name** | Frequency | Percent |
| --- | --- | --- |
| Biscuit chocolade- | 4 | 0,09 |
| Biscuit fourre | 1 | 0,02 |
| Biscuit meergranen- | 2 | 0,05 |
| Biscuit/koekje voor ontbijt | 4 | 0,09 |
| Brood bruin/tarwe verrijkt (vezel/vitamines e.d.) | 37 | 0,86 |
| Brood meergranen- verrijkt (vezel/vitamines e.d.) | 2 | 0,05 |
| Brood wit verrijkt (vezel/vitamines e.d.) | 63 | 1,46 |
| Brood, verrijkt (vezel/vitamines e.d.), n.s. | 2 | 0,05 |
| Cacaopoeder | 49 | 1,14 |
| Cornflakes | 145 | 3,36 |
| Cracker luchtige gewone (wit) | 1 | 0,02 |
| Cracker luchtige volkoren- | 6 | 0,14 |
| Cracker luchtige, n.s. | 5 | 0,12 |
| Cracker overige volkoren | 1 | 0,02 |
| Diksap onverdund | 1 | 0,02 |
| Drank ACE drink | 3 | 0,07 |
| Drank Taksi/Djoezz en soortgelijke | 1 | 0,02 |
| Drank energie- (met cafeine) | 173 | 4,01 |
| Drank sport- energy | 12 | 0,28 |
| Drank sport- isotone | 17 | 0,39 |
| Drank sport-, n.s. | 10 | 0,23 |
| Drank vruchten- 1 vrucht (geen 100% sap) | 9 | 0,21 |
| Drank vruchten- gemengd (geen 100% sap) | 287 | 6,65 |
| Drinkontbijt op yoghurtbasis | 12 | 0,28 |
| Drinkontbijt, n.s. | 2 | 0,05 |
| Evergreen en soortgelijke zoet | 114 | 2,64 |
| Frisdrank Dubbelfrisss en soortgelijke | 7 | 0,16 |
| Frisdrank met vruchtensap | 46 | 1,07 |
| Frisdrank sinas | 6 | 0,14 |
| Frisdrank vruchtensmaak Crystal Clear en soortgelijke | 10 | 0,23 |
| Frisdrank, n.s. | 8 | 0,19 |
| Fruitreepje/-stick voor kinderen | 13 | 0,30 |
| Koek ontbijt- gevuld of met smaakje | 2 | 0,05 |
| Koek ontbijt- volkoren ongevuld | 1 | 0,02 |
| Koekje gewoon zonder creme/glazuur | 1 | 0,02 |
| Koekje kinder- | 3 | 0,07 |
| Koekje met creme | 1 | 0,02 |
| Koekje met yoghurt/melklaagje | 4 | 0,09 |
| Koekje, n.s. | 2 | 0,05 |
| Kwark gewone | 29 | 0,67 |
| Kwark, n.s. | 1 | 0,02 |
| Kwarkyoghurt | 5 | 0,12 |
| Liga en soortgelijke biscuit continue | 2 | 0,05 |
| Liga en soortgelijke fruitkick/haverkick | 11 | 0,25 |
| Liga en soortgelijke milkbreak | 101 | 2,34 |
| Liga en soortgelijke normaal | 4 | 0,09 |
| Liga en soortgelijke, n.s. | 1 | 0,02 |
| Melk chocolade- gewone | 20 | 0,46 |
| Melkdrank geen chocolade/anijs | 4 | 0,09 |
| Minidrink zuivel op yoghurtbasis | 20 | 0,46 |
| Muesli krokante | 6 | 0,14 |
| Muesli ongeroosterd | 2 | 0,05 |
| Muesli, n.s. | 1 | 0,02 |
| Mueslireep gewoon | 8 | 0,19 |
| Mueslireep met yoghurtlaagje | 1 | 0,02 |
| Ontbijtgraan flakes andere dan cornflakes, n.s. | 29 | 0,67 |
| Ontbijtgraan krispies/pops | 48 | 1,11 |
| Ontbijtgraan kussentje | 12 | 0,28 |
| Ontbijtgraan loops/rondjes | 41 | 0,95 |
| Ontbijtgraan voor pap | 5 | 0,12 |
| Ontbijtgraan, n.s. | 5 | 0,12 |
| Ovomaltine (cacaopoeder) | 1 | 0,02 |
| Pudding yoghurt- (niet luchtig) | 1 | 0,02 |
| Sap vruchten en/of groente- concentraat | 1 | 0,02 |
| Sap vruchten- 1 vrucht (100% sap) | 3 | 0,07 |
| Sap vruchten- gemengd (100% sap) | 47 | 1,09 |
| Sap vruchten/groente- | 1 | 0,02 |
| Sap/drank vruchten-, n.s. | 3 | 0,07 |
| Siroop limonade- gewone (vruchtenbasis) | 1650 | 38,24 |
| Siroop limonade- gewone (vruchtenbasis) verdund verhouding onbekend | 755 | 17,50 |
| Siroop limonade- rozenbottel speciale | 27 | 0,63 |
| Siroop limonade- rozenbottel speciale verdund verhouding onbekend | 1 | 0,02 |
| Siroop limonade- rozenbottel vruchtenmix | 39 | 0,90 |
| Siroop limonade- rozenbottel vruchtenmix verdund | 12 | 0,28 |
| Siroop limonade- rozenbottel, n.s. | 33 | 0,76 |
| Siroop limonade-, n.s. | 33 | 0,76 |
| Siroop limonade-, n.s. verdund verhouding onbekend | 82 | 1,90 |
| Sultana fruitbiscuit en soortgelijke | 3 | 0,07 |
| Time Out granenbiscuit en soortgelijke | 1 | 0,02 |
| Vegetarische (ham)burger ongepaneerd | 1 | 0,02 |
| Vegetarische schnitzel/filet/schijf/carre gepaneerd | 6 | 0,14 |
| Vla gewone | 32 | 0,74 |
| Vla, n.s. | 1 | 0,02 |
| Water vitamine- en soortgelijke | 52 | 1,21 |
| Yoghurt Griekse (ingedikte yoghurt) | 7 | 0,16 |
| Yoghurt drink- | 37 | 0,86 |
| Yoghurt gewone (koe) | 60 | 1,39 |
| Yoghurt, n.s. | 2 | 0,05 |
| ZZZ-OUD NIET GEBRUIKEN Yoghurt & Vla kant-en-klaar | 1 | 0,02 |

### Total folate

| **Name** | Frequency | Percent |
| --- | --- | --- |
| Biscuit/koekje voor ontbijt | 4 | 1,04 |
| Cacaopoeder | 49 | 12,76 |
| Cornflakes | 138 | 35,94 |
| Drinkontbijt op yoghurtbasis | 12 | 3,13 |
| Drinkontbijt, n.s. | 2 | 0,52 |
| Frisdrank met vruchtensap | 2 | 0,52 |
| Frisdrank, n.s. | 3 | 0,78 |
| Gistextract Marmite en soortgelijke | 19 | 4,95 |
| Koekje gewoon zonder creme/glazuur | 1 | 0,26 |
| Koekje kinder- | 1 | 0,26 |
| Liga en soortgelijke biscuit continue | 1 | 0,26 |
| Melk chocolade- gewone | 20 | 5,21 |
| Muesli krokante | 6 | 1,56 |
| Muesli ongeroosterd | 2 | 0,52 |
| Muesli, n.s. | 1 | 0,26 |
| Mueslireep gewoon | 5 | 1,30 |
| Mueslireep met yoghurtlaagje | 1 | 0,26 |
| Ontbijtgraan flakes andere dan cornflakes, n.s. | 25 | 6,51 |
| Ontbijtgraan krispies/pops | 48 | 12,50 |
| Ontbijtgraan kussentje | 12 | 3,13 |
| Ontbijtgraan loops/rondjes | 24 | 6,25 |
| Ontbijtgraan, n.s. | 5 | 1,30 |
| Ovomaltine (cacaopoeder) | 1 | 0,26 |
| Sap vruchten- gemengd (100% sap) | 1 | 0,26 |
| Sap vruchten/groente- | 1 | 0,26 |

### Vitamin B12

| **Name** | Frequency | Percent |
| --- | --- | --- |
| Amandeldrank Alpro en soortgelijke | 28 | 1,69 |
| Brood meergranen- verrijkt (vezel/vitamines e.d.) | 7 | 0,42 |
| Brood, n.s. | 1 | 0,06 |
| Cornflakes | 145 | 8,73 |
| Drank energie- (met cafeine) | 167 | 10,05 |
| Drank vruchten- 1 vrucht (geen 100% sap) | 9 | 0,54 |
| Drank vruchten- gemengd (geen 100% sap) | 242 | 14,57 |
| Drinkontbijt op yoghurtbasis | 13 | 0,78 |
| Drinkontbijt, n.s. | 2 | 0,12 |
| Falafel | 6 | 0,36 |
| Frisdrank Dubbelfrisss en soortgelijke | 5 | 0,30 |
| Frisdrank met vruchtensap | 37 | 2,23 |
| Frisdrank sinas | 6 | 0,36 |
| Frisdrank vruchtensmaak Crystal Clear en soortgelijke | 7 | 0,42 |
| Frisdrank, n.s. | 2 | 0,12 |
| Gistextract Marmite en soortgelijke | 19 | 1,14 |
| Haverdrank Alpro en soortgelijke | 2 | 0,12 |
| Koek ontbijt- gevuld of met smaakje | 2 | 0,12 |
| Koek ontbijt- volkoren ongevuld | 1 | 0,06 |
| Koekje kinder- | 3 | 0,18 |
| Kokosnootdrank Alpro en soortgelijke | 2 | 0,12 |
| Kwark gewone | 29 | 1,75 |
| Kwark, n.s. | 1 | 0,06 |
| Kwarkyoghurt | 5 | 0,30 |
| Melk, n.s. | 2 | 0,12 |
| Melkdrank geen chocolade/anijs | 56 | 3,37 |
| Muesli krokante | 6 | 0,36 |
| Muesli ongeroosterd | 2 | 0,12 |
| Muesli, n.s. | 1 | 0,06 |
| Mueslireep gewoon | 5 | 0,30 |
| Mueslireep met yoghurtlaagje | 1 | 0,06 |
| Ontbijtgraan flakes andere dan cornflakes, n.s. | 29 | 1,75 |
| Ontbijtgraan krispies/pops | 48 | 2,89 |
| Ontbijtgraan kussentje | 12 | 0,72 |
| Ontbijtgraan loops/rondjes | 41 | 2,47 |
| Ontbijtgraan, n.s. | 5 | 0,30 |
| Ovomaltine (cacaopoeder) | 1 | 0,06 |
| Pudding yoghurt- (niet luchtig) | 1 | 0,06 |
| Rijstdrank Ricedream en soortgelijke | 22 | 1,32 |
| Sap vruchten en/of groente- concentraat | 1 | 0,06 |
| Sap vruchten- 1 vrucht (100% sap) | 3 | 0,18 |
| Sap vruchten- gemengd (100% sap) | 41 | 2,47 |
| Sap vruchten/groente- | 1 | 0,06 |
| Sap/drank vruchten-, n.s. | 3 | 0,18 |
| Sojadessert | 17 | 1,02 |
| Sojadrink/melk | 216 | 13,00 |
| Sojavla | 16 | 0,96 |
| Sojayoghurt | 62 | 3,73 |
| Vegetarisch broodbeleg boterhamworst | 6 | 0,36 |
| Vegetarisch broodbeleg filet americain | 2 | 0,12 |
| Vegetarisch broodbeleg ham | 5 | 0,30 |
| Vegetarisch broodbeleg smeerworst/pate | 24 | 1,44 |
| Vegetarisch broodbeleg, n.s. | 1 | 0,06 |
| Vegetarisch gehakt | 22 | 1,32 |
| Vegetarische (ham)burger gepaneerd | 10 | 0,60 |
| Vegetarische (ham)burger ongepaneerd | 20 | 1,20 |
| Vegetarische bal/balletjes | 17 | 1,02 |
| Vegetarische reepjes/roerbakblokjes/stukjes | 7 | 0,42 |
| Vegetarische schnitzel/filet/schijf/carre gepaneerd | 12 | 0,72 |
| Vegetarische schnitzel/filet/schijf/carre ongepaneerd | 4 | 0,24 |
| Vegetarische worst rook-/braad-/knak- | 9 | 0,54 |
| Vla gewone | 32 | 1,93 |
| Vla, n.s. | 1 | 0,06 |
| Water vitamine- en soortgelijke | 52 | 3,13 |
| Yoghurt Griekse (ingedikte yoghurt) | 7 | 0,42 |
| Yoghurt drink- | 34 | 2,05 |
| Yoghurt gewone (koe) | 60 | 3,61 |
| Yoghurt, n.s. | 2 | 0,12 |
| ZZZ-OUD NIET GEBRUIKEN Yoghurt & Vla kant-en-klaar | 1 | 0,06 |

### Vitamin C

| name | Frequency | Percent |
| --- | --- | --- |
| Cacaopoeder | 49 | 0,90 |
| Cornflakes | 10 | 0,18 |
| Diksap onverdund | 4 | 0,07 |
| Diksap verdund verhouding onbekend | 1 | 0,02 |
| Drank ACE drink | 23 | 0,42 |
| Drank Taksi/Djoezz en soortgelijke | 224 | 4,13 |
| Drank aloe vera | 7 | 0,13 |
| Drank energie- (met cafeine) | 4 | 0,07 |
| Drank sport- energy | 9 | 0,17 |
| Drank sport-, n.s. | 4 | 0,07 |
| Drank vruchten- 1 vrucht (geen 100% sap) | 404 | 7,45 |
| Drank vruchten- gemengd (geen 100% sap) | 658 | 12,14 |
| Drank vruchten- met water (ongezoet) | 11 | 0,20 |
| Drinkontbijt op fruitbasis | 2 | 0,04 |
| Drinkontbijt op yoghurtbasis | 1 | 0,02 |
| Frisdrank Dubbelfrisss en soortgelijke | 92 | 1,70 |
| Frisdrank Rivella en soortgelijke | 5 | 0,09 |
| Frisdrank Spa Fruit en soortgelijke | 34 | 0,63 |
| Frisdrank appel | 5 | 0,09 |
| Frisdrank icetea/ijsthee | 2 | 0,04 |
| Frisdrank met vruchtensap | 233 | 4,30 |
| Frisdrank sinas | 29 | 0,54 |
| Frisdrank vruchtensmaak Crystal Clear en soortgelijke | 10 | 0,18 |
| Frisdrank, n.s. | 16 | 0,30 |
| IJs aan stokje (melkbasis) met waterijs | 1 | 0,02 |
| IJs aan stokje waterijs | 44 | 0,81 |
| Jam gewone/extra | 20 | 0,37 |
| Melk chocolade- gewone | 20 | 0,37 |
| Melk, n.s. | 2 | 0,04 |
| Melkdrank geen chocolade/anijs | 56 | 1,03 |
| Minidrink fruitbasis (100% sap) | 1 | 0,02 |
| Minidrink zuivel op yoghurtbasis | 8 | 0,15 |
| Muesli ongeroosterd | 1 | 0,02 |
| Muesli, n.s. | 1 | 0,02 |
| Mueslireep gewoon | 1 | 0,02 |
| Ontbijtgraan flakes andere dan cornflakes, n.s. | 2 | 0,04 |
| Ontbijtgraan voor pap | 5 | 0,09 |
| Ovomaltine (cacaopoeder) | 1 | 0,02 |
| Sap appel- (100% sap) | 65 | 1,20 |
| Sap bessen- (100% sap) | 3 | 0,06 |
| Sap groente-, n.s. | 1 | 0,02 |
| Sap tomaten- | 2 | 0,04 |
| Sap tomaten-groente | 6 | 0,11 |
| Sap vruchten en/of groente- concentraat | 2 | 0,04 |
| Sap vruchten- 1 vrucht (100% sap) | 33 | 0,61 |
| Sap vruchten- gemengd (100% sap) | 61 | 1,13 |
| Sap vruchten/groente- | 7 | 0,13 |
| Sap wortel- | 2 | 0,04 |
| Sap/drank vruchten-, n.s. | 29 | 0,54 |
| Siroop limonade- gewone (vruchtenbasis) | 1892 | 34,91 |
| Siroop limonade- gewone (vruchtenbasis) verdund verhouding onbekend | 800 | 14,76 |
| Siroop limonade- rozenbottel speciale | 49 | 0,90 |
| Siroop limonade- rozenbottel speciale verdund verhouding onbekend | 12 | 0,22 |
| Siroop limonade- rozenbottel vruchtenmix | 127 | 2,34 |
| Siroop limonade- rozenbottel vruchtenmix verdund | 56 | 1,03 |
| Siroop limonade- rozenbottel, n.s. | 35 | 0,65 |
| Siroop limonade- rozenbottel, n.s. verdund verhouding onbekend | 4 | 0,07 |
| Siroop limonade-, n.s. | 37 | 0,68 |
| Siroop limonade-, n.s. verdund verhouding onbekend | 87 | 1,61 |
| Sojadrink/melk | 12 | 0,22 |
| Water vitamine- en soortgelijke | 59 | 1,09 |
| Yoghurt drink- | 36 | 0,66 |
| Yoghurt gewone (koe) | 3 | 0,06 |

###

### Vitamin D

| name | Frequency | Percent |
| --- | --- | --- |
| Amandeldrank Alpro en soortgelijke | 28 | 3,89 |
| Cacaopoeder | 49 | 6,82 |
| Cornflakes | 65 | 9,04 |
| Dessert kindertoetje | 38 | 5,29 |
| Drinkontbijt, n.s. | 1 | 0,14 |
| Haverdrank Alpro en soortgelijke | 2 | 0,28 |
| Kokosnootdrank Alpro en soortgelijke | 2 | 0,28 |
| Kwark gewone | 72 | 10,01 |
| Kwark, n.s. | 4 | 0,56 |
| Kwarkyoghurt | 11 | 1,53 |
| Melkdrank geen chocolade/anijs | 3 | 0,42 |
| Minidrink zuivel op yoghurtbasis | 20 | 2,78 |
| Ontbijtgraan flakes andere dan cornflakes, n.s. | 6 | 0,83 |
| Ontbijtgraan krispies/pops | 31 | 4,31 |
| Ontbijtgraan kussentje | 1 | 0,14 |
| Ontbijtgraan loops/rondjes | 22 | 3,06 |
| Ontbijtgraan, n.s. | 2 | 0,28 |
| Rijstdrank Ricedream en soortgelijke | 22 | 3,06 |
| Sojadessert | 17 | 2,36 |
| Sojadrink/melk | 193 | 26,84 |
| Sojavla | 16 | 2,23 |
| Sojayoghurt | 62 | 8,62 |
| Yoghurt drink- | 48 | 6,68 |
| Yoghurt gewone (koe) | 1 | 0,14 |
| ZZZ-OUD NIET GEBRUIKEN Yoghurt & Vla kant-en-klaar | 3 | 0,42 |

### Vitamin E

| name | Frequency | Percent |
| --- | --- | --- |
| Amandeldrank Alpro en soortgelijke | 28 | 0,84 |
| Bak-en-braadvet | 4 | 0,12 |
| Biscuit meergranen- | 1 | 0,03 |
| Biscuit volkoren- | 1 | 0,03 |
| Biscuit/koekje voor ontbijt | 10 | 0,30 |
| Cornflakes | 18 | 0,54 |
| Diksap onverdund | 1 | 0,03 |
| Drank ACE drink | 25 | 0,75 |
| Drank Taksi/Djoezz en soortgelijke | 17 | 0,51 |
| Drank energie- (met cafeine) | 2 | 0,06 |
| Drank sport- energy | 3 | 0,09 |
| Drank sport- isotone | 10 | 0,30 |
| Drank sport-, n.s. | 6 | 0,18 |
| Drank vruchten- 1 vrucht (geen 100% sap) | 15 | 0,45 |
| Drank vruchten- gemengd (geen 100% sap) | 324 | 9,67 |
| Drank vruchten- met water (ongezoet) | 1 | 0,03 |
| Drinkontbijt op fruitbasis | 2 | 0,06 |
| Frisdrank Dubbelfrisss en soortgelijke | 10 | 0,30 |
| Frisdrank met vruchtensap | 62 | 1,85 |
| Frisdrank sinas | 5 | 0,15 |
| Frisdrank vruchtensmaak Crystal Clear en soortgelijke | 2 | 0,06 |
| Haverdrank Alpro en soortgelijke | 2 | 0,06 |
| Koek muesli- | 1 | 0,03 |
| Koek ontbijt- gevuld of met smaakje | 2 | 0,06 |
| Koek ontbijt- volkoren ongevuld | 1 | 0,03 |
| Koekje gewoon zonder creme/glazuur | 1 | 0,03 |
| Koekje, n.s. | 1 | 0,03 |
| Liga en soortgelijke biscuit continue | 1 | 0,03 |
| Melk chocolade- gewone | 20 | 0,60 |
| Mueslireep gewoon | 2 | 0,06 |
| Olie dieet- (cholesterolverlagend) | 26 | 0,78 |
| Ontbijtgraan voor pap | 5 | 0,15 |
| Ovomaltine (cacaopoeder) | 1 | 0,03 |
| Rijstdrank Ricedream en soortgelijke | 4 | 0,12 |
| Sap bessen- (100% sap) | 2 | 0,06 |
| Sap vruchten en/of groente- concentraat | 1 | 0,03 |
| Sap vruchten- 1 vrucht (100% sap) | 8 | 0,24 |
| Sap vruchten- gemengd (100% sap) | 55 | 1,64 |
| Sap vruchten/groente- | 1 | 0,03 |
| Sap/drank vruchten-, n.s. | 7 | 0,21 |
| Siroop limonade- gewone (vruchtenbasis) | 1650 | 49,24 |
| Siroop limonade- gewone (vruchtenbasis) verdund verhouding onbekend | 755 | 22,53 |
| Siroop limonade- rozenbottel speciale | 27 | 0,81 |
| Siroop limonade- rozenbottel speciale verdund verhouding onbekend | 1 | 0,03 |
| Siroop limonade- rozenbottel vruchtenmix | 39 | 1,16 |
| Siroop limonade- rozenbottel vruchtenmix verdund | 13 | 0,39 |
| Siroop limonade- rozenbottel, n.s. | 33 | 0,98 |
| Siroop limonade-, n.s. | 33 | 0,98 |
| Siroop limonade-, n.s. verdund verhouding onbekend | 82 | 2,45 |
| Sojadrink/melk | 29 | 0,87 |
| Time Out granenbiscuit en soortgelijke | 1 | 0,03 |

### Calcium

| name | Frequency | Percent |
| --- | --- | --- |
| Amandeldrank Alpro en soortgelijke | 28 | 1,92 |
| Biscuit chocolade- | 4 | 0,27 |
| Biscuit fourre | 1 | 0,07 |
| Biscuit, n.s. | 1 | 0,07 |
| Biscuit/koekje voor ontbijt | 1 | 0,07 |
| Cornflakes | 35 | 2,40 |
| Cracker overige volkoren | 10 | 0,69 |
| Dessert kindertoetje | 38 | 2,61 |
| Drank vruchten- gemengd (geen 100% sap) | 1 | 0,07 |
| Drank zuivel-, n.s. | 1 | 0,07 |
| Drinkontbijt op yoghurtbasis | 22 | 1,51 |
| Drinkontbijt, n.s. | 3 | 0,21 |
| Evergreen en soortgelijke zoet | 94 | 6,45 |
| Frisdrank met vruchtensap | 2 | 0,14 |
| Frisdrank vruchtensmaak Crystal Clear en soortgelijke | 7 | 0,48 |
| Frisdrank, n.s. | 3 | 0,21 |
| Fruitreepje/-stick voor kinderen | 13 | 0,89 |
| Haverdrank Alpro en soortgelijke | 2 | 0,14 |
| Kaas smeer- | 62 | 4,26 |
| Knackebrod overige (geef specifieke soortnaam op) | 2 | 0,14 |
| Koek eier- (gewone) | 1 | 0,07 |
| Koek eier-, n.s. | 2 | 0,14 |
| Koek ontbijt- gevuld of met smaakje | 2 | 0,14 |
| Koek ontbijt- volkoren ongevuld | 1 | 0,07 |
| Koekje gewoon zonder creme/glazuur | 1 | 0,07 |
| Koekje kinder- | 6 | 0,41 |
| Koekje met creme | 1 | 0,07 |
| Koekje met yoghurt/melklaagje | 10 | 0,69 |
| Koekje zandtaartdeeg met chocola | 1 | 0,07 |
| Koekje, n.s. | 2 | 0,14 |
| Kokosnootdrank Alpro en soortgelijke | 2 | 0,14 |
| Kwark gewone | 73 | 5,01 |
| Kwark, n.s. | 4 | 0,27 |
| Kwarkyoghurt | 11 | 0,75 |
| Liga en soortgelijke biscuit continue | 1 | 0,07 |
| Liga en soortgelijke fruitkick/haverkick | 11 | 0,75 |
| Liga en soortgelijke milkbreak | 102 | 7,00 |
| Liga en soortgelijke normaal | 1 | 0,07 |
| Liga en soortgelijke, n.s. | 1 | 0,07 |
| Melk chocolade- gewone | 20 | 1,37 |
| Melk verrijkte (vit./min. e.d.) | 3 | 0,21 |
| Melk, n.s. | 2 | 0,14 |
| Melkdrank geen chocolade/anijs | 58 | 3,98 |
| Mueslireep gewoon | 4 | 0,27 |
| Mueslireep met yoghurtlaagje | 1 | 0,07 |
| Ontbijtgraan flakes andere dan cornflakes, n.s. | 6 | 0,41 |
| Ontbijtgraan krispies/pops | 30 | 2,06 |
| Ontbijtgraan kussentje | 1 | 0,07 |
| Ontbijtgraan loops/rondjes | 39 | 2,68 |
| Ontbijtgraan, n.s. | 2 | 0,14 |
| Ovomaltine (cacaopoeder) | 1 | 0,07 |
| Pudding yoghurt- (niet luchtig) | 1 | 0,07 |
| Rijstdrank Ricedream en soortgelijke | 22 | 1,51 |
| Sojadessert | 17 | 1,17 |
| Sojadrink/melk | 216 | 14,82 |
| Sojavla | 16 | 1,10 |
| Sojayoghurt | 62 | 4,26 |
| Sultana fruitbiscuit en soortgelijke | 32 | 2,20 |
| Time Out granenbiscuit en soortgelijke | 1 | 0,07 |
| Vegetarische (ham)burger ongepaneerd | 1 | 0,07 |
| Vegetarische schnitzel/filet/schijf/carre gepaneerd | 6 | 0,41 |
| Vla gewone | 53 | 3,64 |
| Vla, n.s. | 1 | 0,07 |
| Water vitamine- en soortgelijke | 52 | 3,57 |
| Yoghurt Griekse (ingedikte yoghurt) | 7 | 0,48 |
| Yoghurt drink- | 173 | 11,87 |
| Yoghurt gewone (koe) | 61 | 4,19 |
| Yoghurt, n.s. | 2 | 0,14 |
| ZZZ-OUD NIET GEBRUIKEN Yoghurt & Vla kant-en-klaar | 4 | 0,27 |

### Iron

| name | Frequency | Percent |
| --- | --- | --- |
| Biscuit chocolade- | 4 | 0,41 |
| Biscuit fourre | 1 | 0,10 |
| Biscuit meergranen- | 3 | 0,31 |
| Biscuit volkoren- | 1 | 0,10 |
| Biscuit/koekje voor ontbijt | 6 | 0,61 |
| Brood bruin/tarwe verrijkt (vezel/vitamines e.d.) | 37 | 3,79 |
| Brood meergranen- verrijkt (vezel/vitamines e.d.) | 9 | 0,92 |
| Brood wit verrijkt (vezel/vitamines e.d.) | 68 | 6,96 |
| Brood, n.s. | 1 | 0,10 |
| Brood, verrijkt (vezel/vitamines e.d.), n.s. | 2 | 0,20 |
| Cacaopoeder | 49 | 5,02 |
| Cornflakes | 141 | 14,43 |
| Cracker luchtige gewone (wit) | 1 | 0,10 |
| Cracker luchtige volkoren- | 6 | 0,61 |
| Cracker luchtige, n.s. | 5 | 0,51 |
| Cracker overige volkoren | 1 | 0,10 |
| Evergreen en soortgelijke zoet | 114 | 11,67 |
| Falafel | 6 | 0,61 |
| Fruitreepje/-stick voor kinderen | 13 | 1,33 |
| Koek muesli- | 1 | 0,10 |
| Koek ontbijt- gevuld of met smaakje | 2 | 0,20 |
| Koek ontbijt- volkoren ongevuld | 1 | 0,10 |
| Koekje kinder- | 3 | 0,31 |
| Koekje met creme | 1 | 0,10 |
| Koekje met yoghurt/melklaagje | 4 | 0,41 |
| Koekje, n.s. | 3 | 0,31 |
| Liga en soortgelijke biscuit continue | 1 | 0,10 |
| Liga en soortgelijke fruitkick/haverkick | 11 | 1,13 |
| Liga en soortgelijke milkbreak | 101 | 10,34 |
| Liga en soortgelijke normaal | 4 | 0,41 |
| Liga en soortgelijke, n.s. | 1 | 0,10 |
| Muesli krokante | 6 | 0,61 |
| Muesli ongeroosterd | 2 | 0,20 |
| Muesli, n.s. | 1 | 0,10 |
| Mueslireep gewoon | 8 | 0,82 |
| Mueslireep met yoghurtlaagje | 1 | 0,10 |
| Ontbijtgraan flakes andere dan cornflakes, n.s. | 29 | 2,97 |
| Ontbijtgraan koekvorm Weetabix en soortgelijke | 4 | 0,41 |
| Ontbijtgraan krispies/pops | 48 | 4,91 |
| Ontbijtgraan kussentje | 12 | 1,23 |
| Ontbijtgraan loops/rondjes | 41 | 4,20 |
| Ontbijtgraan voor pap | 7 | 0,72 |
| Ontbijtgraan, n.s. | 5 | 0,51 |
| Siroop limonade- rozenbottel speciale | 4 | 0,41 |
| Siroop limonade- rozenbottel vruchtenmix | 1 | 0,10 |
| Sojadrink/melk | 12 | 1,23 |
| Stroop appel- | 44 | 4,50 |
| Sultana fruitbiscuit en soortgelijke | 3 | 0,31 |
| Time Out granenbiscuit en soortgelijke | 2 | 0,20 |
| Vegetarisch broodbeleg boterhamworst | 6 | 0,61 |
| Vegetarisch broodbeleg filet americain | 2 | 0,20 |
| Vegetarisch broodbeleg ham | 5 | 0,51 |
| Vegetarisch broodbeleg smeerworst/pate | 24 | 2,46 |
| Vegetarisch broodbeleg, n.s. | 1 | 0,10 |
| Vegetarisch gehakt | 22 | 2,25 |
| Vegetarische (ham)burger gepaneerd | 10 | 1,02 |
| Vegetarische (ham)burger ongepaneerd | 21 | 2,15 |
| Vegetarische bal/balletjes | 17 | 1,74 |
| Vegetarische reepjes/roerbakblokjes/stukjes | 7 | 0,72 |
| Vegetarische schnitzel/filet/schijf/carre gepaneerd | 18 | 1,84 |
| Vegetarische schnitzel/filet/schijf/carre ongepaneerd | 4 | 0,41 |
| Vegetarische worst rook-/braad-/knak- | 9 | 0,92 |

### Magnesium

| name | Frequency | Percent |
| --- | --- | --- |
| Biscuit meergranen- | 1 | 1,08 |
| Biscuit volkoren- | 1 | 1,08 |
| Biscuit/koekje voor ontbijt | 10 | 10,75 |
| Cacaopoeder | 49 | 52,69 |
| Frisdrank met vruchtensap | 2 | 2,15 |
| Frisdrank, n.s. | 3 | 3,23 |
| Koek muesli- | 1 | 1,08 |
| Koekje gewoon zonder creme/glazuur | 1 | 1,08 |
| Koekje, n.s. | 1 | 1,08 |
| Liga en soortgelijke biscuit continue | 1 | 1,08 |
| Melk chocolade- gewone | 20 | 21,51 |
| Mueslireep gewoon | 1 | 1,08 |
| Ovomaltine (cacaopoeder) | 1 | 1,08 |
| Time Out granenbiscuit en soortgelijke | 1 | 1,08 |
