# Supplementary material for: Contribution of voluntary fortified foods to micronutrient intake in The Netherlands
Source: Eur J Nutr. 2022 Jan 1;61(3):1649–63. doi: 10.1007/s00394-021-02728-4 (PMC8921121; doi:10.1007/s00394-021-02728-4)
Supplement: Supplementary file 3 — Supplementary file3 (DOCX 27 kb) [file 394_2021_2728_MOESM3_ESM.docx]

Appendix 3 – The assessment of excessive micronutrient intakes separate for users and non-users of fortified fats

| **Micronutrient** | **Age** | **Gender** | **UL** | **Non-users** |  | **Users** |
| --- | --- | --- | --- | --- | --- | --- |
|  |  |  |  | **%>UL** |  | **%>UL** |
| **Vitamin D (µg)** | 1-17 | Boys | 50/100 | 0 (0-0) |  | 0 (0-0) |
|  |  | Girls |  | 0 (0-0) |  | 0 (0-0) |
|  | 18-79 | Men | 100 | 0 (0-0) |  | 0 (0-0) |
|  |  | Women |  | 0 (0-0) |  | 0 (0-0) |
| **Vitamin B6 (mg)** | 1-17 | Boys | 5/7/10/15/20 | 0 (0-0) |  | 0 (0-0) |
|  |  | Girls |  | 0 (0-0) |  | 0 (0-0) |
|  | 18-79 | Men | 25 | 0 (0-0) |  | 0 (0-0) |
|  |  | Women |  | 0 (0-0) |  | 0 (0-0) |
| **Vitamin E (mg)** | 1-17 | Boys | 100/120/160/220/260 | 0 (0-0) |  | 0 (0-0) |
|  |  | Girls |  | 0 (0-0) |  | 0 (0-0) |
|  | 18-79 | Men | 300 | 0 (0-0) |  | 0 (0-0) |
|  |  | Women |  | 0 (0-0) |  | 0 (0-0) |
| **Calcium (mg)** | 1-17 | Boys | - | - |  | - |
|  |  | Girls |  |  |  |  |
|  | 18-79 | Men | 2500 | 0.2 (0.1-0.3) |  | 0.1 (0-0.3) |
|  |  | Women |  | 0 (0-0) |  | 0 (0-0) |
